# Supplementary material for: SUMO2 conjugation of PCNA facilitates chromatin remodeling to resolve transcription-replication conflicts
Source: Nat Commun. 2018 Jul 13;9:2706. doi: 10.1038/s41467-018-05236-y (PMC6045570; doi:10.1038/s41467-018-05236-y)
Supplement: Supplementary file 1 — Supplementary Information [file 41467_2018_5236_MOESM1_ESM.pdf]

SUMO2 conjugation of PCNA facilitates chromatin remodeling to resolve transcription-replication conflicts

Supplementary Information

Li et al

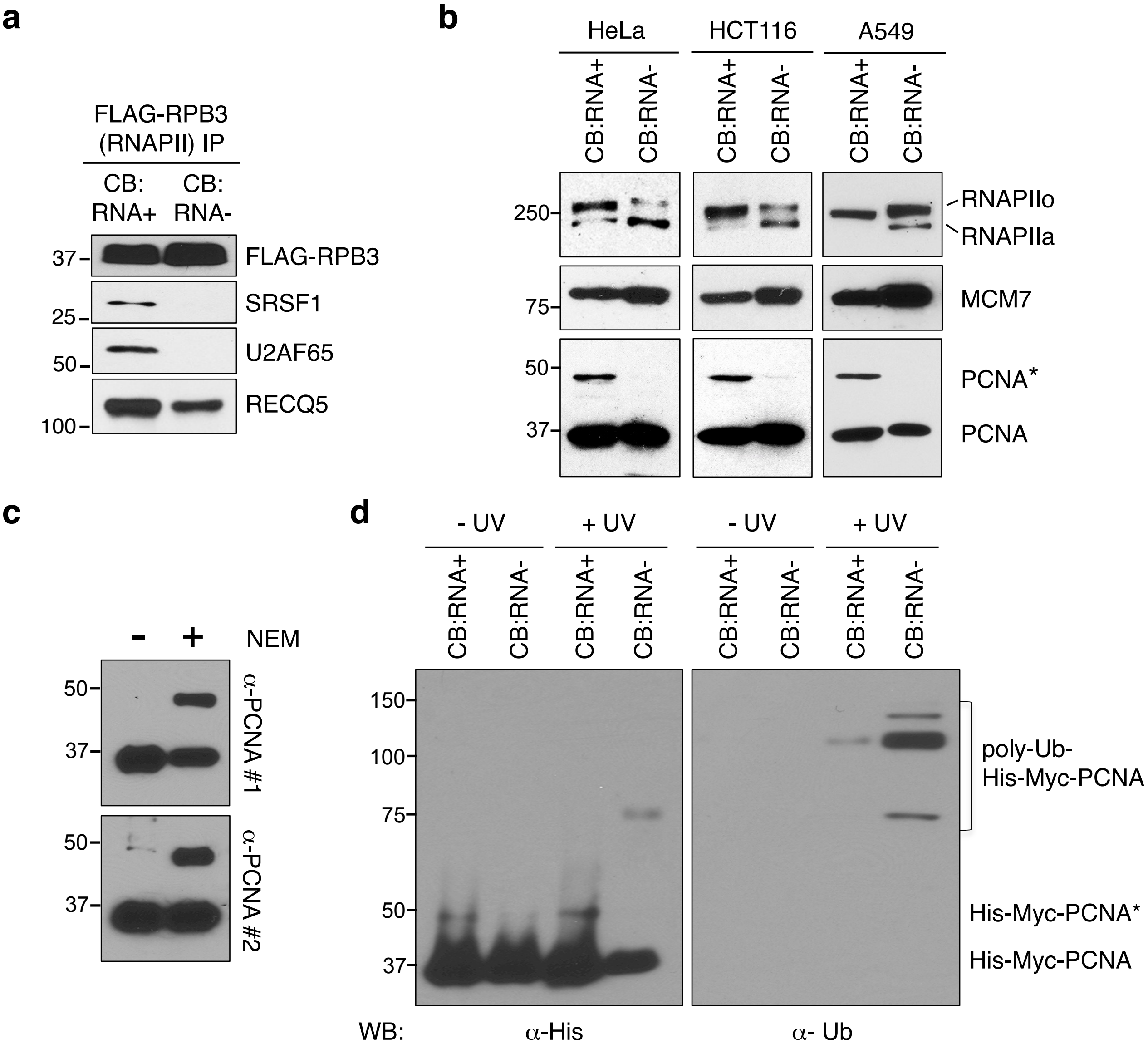

Supplementary Figure 1. DNA damage-independent PCNA modification in the CB:RNA+ fraction. (a) Western blot analysis of FLAG-RPB3, SRSF1, U2AF65, and RECQ5 in RNAPII complexes immunopurified using FLAG-RPB3 from the CB:RNA+ and CB:RNA- fractions of HEK293T cells expressing FLAG-RPB3. (b) Western blot analysis of the indicated proteins in CB:RNA+ and CB:RNA- fractions prepared from HeLa, HCT116, and A549 cells. The post-translationally modified form of PCNA is indicated with an asterisk (*). (c) Western blot analysis of PCNA in CB:RNA+ fractions prepared from HEK293T cells in the presence or absence of N-ethylmaleimide (NEM), an inhibitor of deubiquitinases and SUMO hydrolases. (d) Western blot analysis of His-Myc-PCNA purified using Ni-NTA under denaturing conditions from the CB:RNA+ and CB:RNA- fractions prepared from HEK293T cells transfected with a His-Myc-PCNA construct and treated with or without UV radiation. The same membrane was first probed with an antibody specific to ubiquitin (Ub), followed by an α-His antibody after stripping.

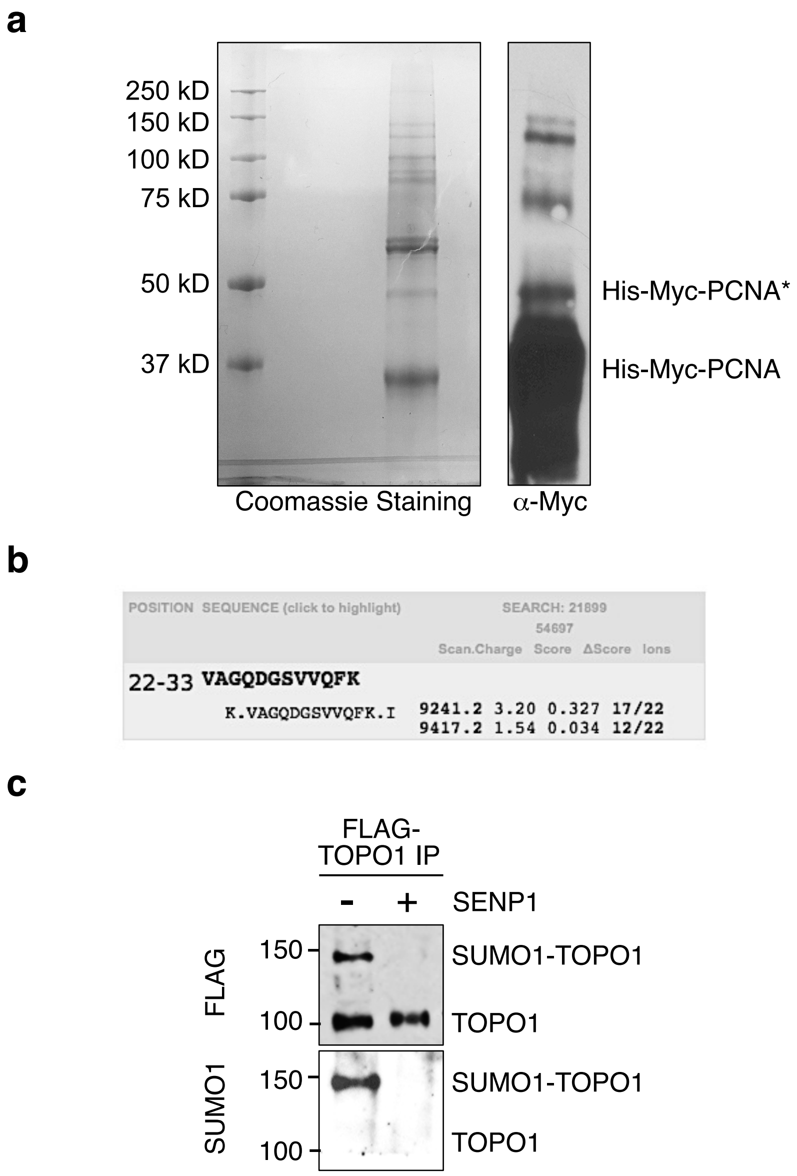

Supplementary Figure 2. Identification of SUMO2 conjugation of PCNA in the CB:RNA+ fraction. (a) Coomassie blue stained gel (left) and western blot analysis using an α-Myc antibody (right) of His-Myc-PCNA purified using Ni-NTA under denaturing conditions from the CB:RNA+ fraction of HEK293T cells transfected with a His-Myc-PCNA construct. The post-translationally modified form of PCNA is indicated with an asterisk (*). (b) The peptide sequence corresponding to SUMO2/3 identified by mass spectrometry analysis of the purified His-Myc-PCNA* band excised from the Coomassie blue stained SDS-PAGE gel shown in (a). (c) Western blot analysis of the purified non-modified and SUMO1-conjugated FLAG-TOPO1 prepared from the CB:RNA+ of cells expressing FLAG-TOPO1. The blots were probed using an α-FLAG antibody. The purified FLAG-TOPO1 was treated with the mammalian SUMO-specific protease SENP1 to confirm the SUMO1 conjugation.

a

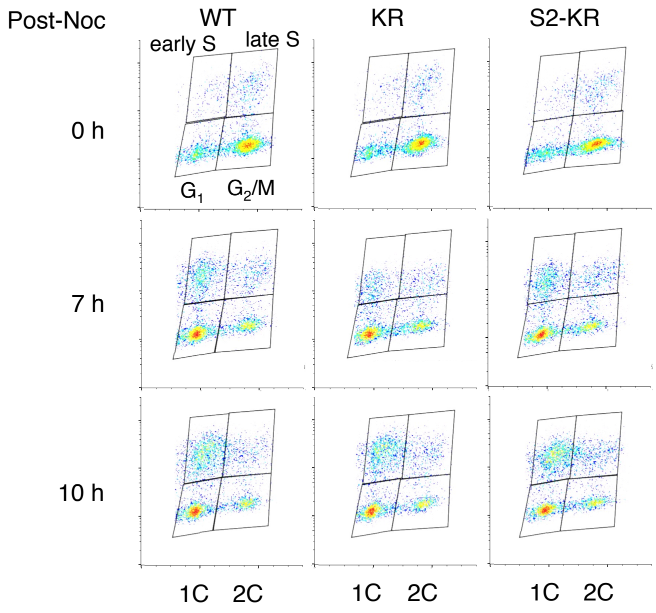

b

	0 h			7 h			10 h		
	**WT**	**KR**	**S2-KR**	**WT**	**KR**	**S2-KR**	**WT**	**KR**	**S2-KR**
**G_2/M**	59.4 %	58.8 %	58.0 %	20.4 %	26.5 %	22.6 %	17.9 %	18.8 %	17.6 %
**G_1**	19.0 %	19.4 %	19.8 %	40.9 %	44.6 %	40.3 %	33.1 %	34.7 %	30.6 %
**early S**	6.1 %	6.8 %	6.5 %	20.8 %	14.5 %	20.3 %	31.1 %	29.7 %	31.5 %
**late S**	13.2 %	13.3 %	13.4 %	13.3%	11.9 %	13.8 %	13.7 %	13.5 %	17.1 %

c

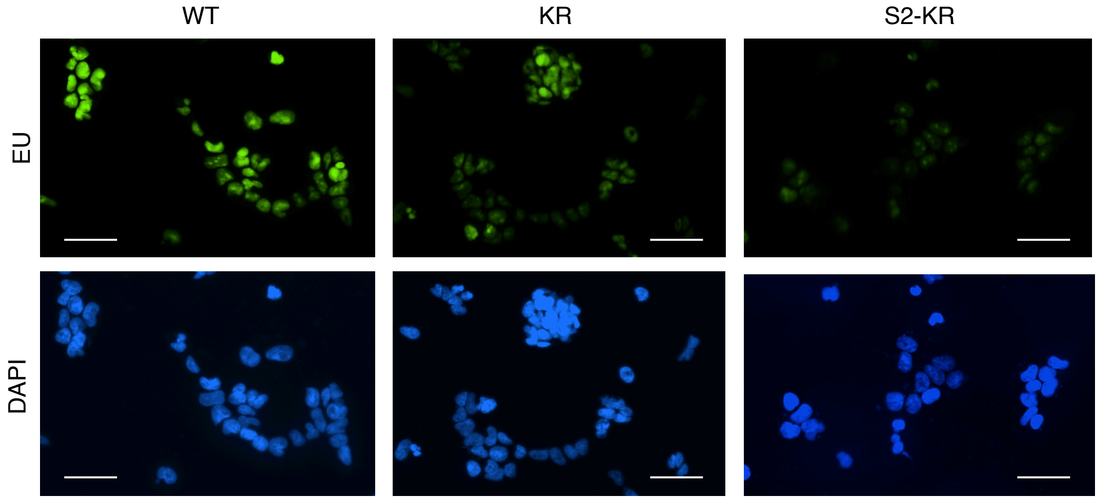

Supplementary Figure 3. SUMO2-PCNA affects cell cycle progression and transcription efficiency. (a) and (b) Flow cytometry to determine cell cycle distribution of HEK293T cells overexpressing WT, KR, or S2-KR FLAG-PCNA after release from nocodazole (Noc) blocking at the indicated time points. 1C and 2C represent cells containing one or two copies of each chromosome, respectively. (a) The cells were labeled with BrdU and co-stained for BrdU incorporation (Y-axis) and DNA content (propidium iodide [PI], X-axis). Percentages of cells in G_2/M, G_1, early S, and late S phases are shown in (b). (c) Representative fluorescence images of 5'-Ethynyl Uridine (EU) incorporation in HEK293T cells overexpressing PCNA WT, KR, or S2-KR. Nuclei were counterstained with DAPI. Scale bar, 50 µm.

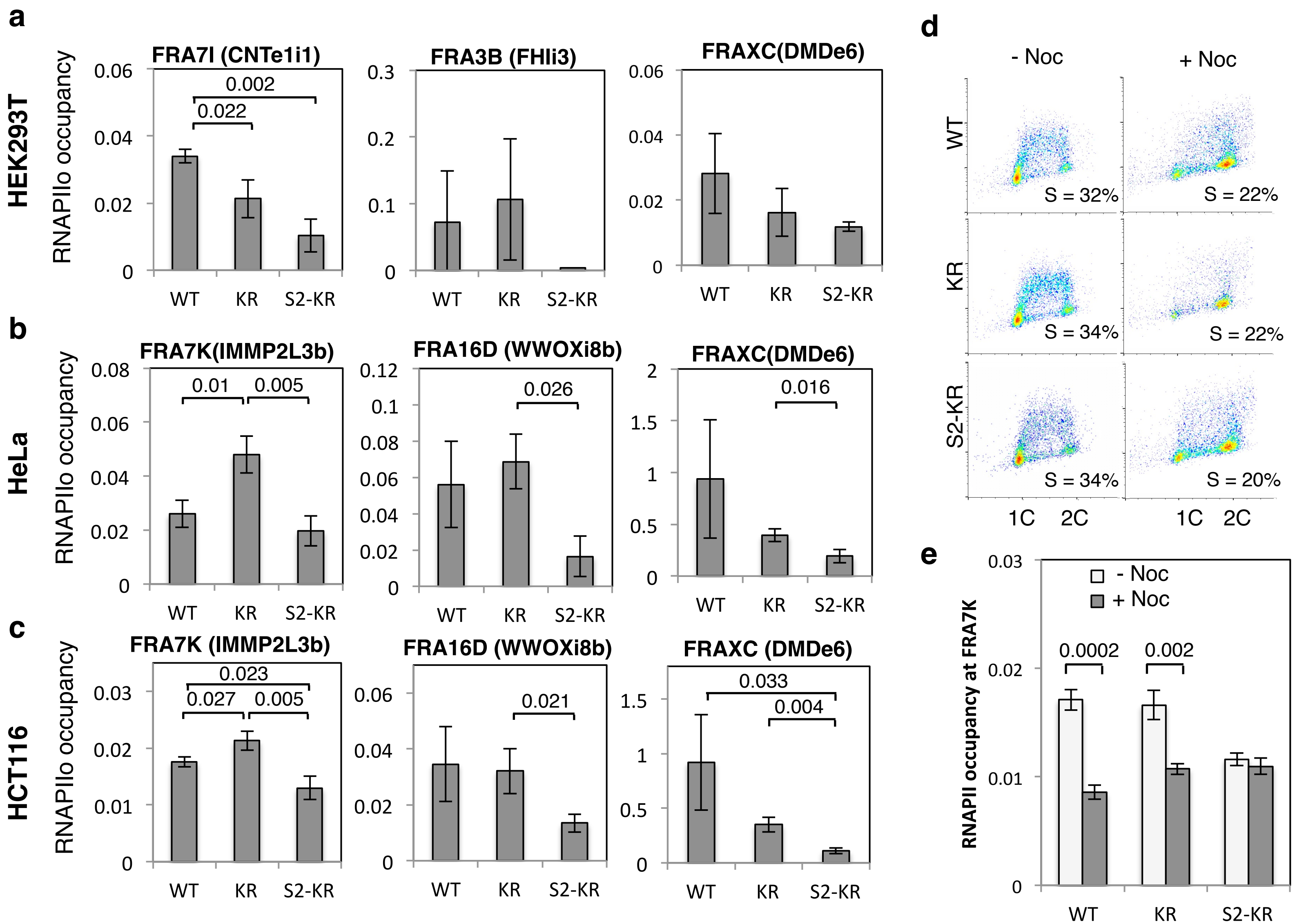

Supplementary Figure 4. SUMO2-PCNA reduces RNAPII occupancy at CFSs. (a)-(c) ChIP analysis of RNAPIIo occupancy at the indicated CFSs using primers derived from Helmrich et al (Supplementary Table 2) in HEK293T (a), HeLa (b), and HCT116 (c) cells overexpressing WT, KR or S2-KR FLAG-PCNA. (d) Flow cytometry to determine cell cycle distribution of HEK293T cells overexpressing WT, KR or S2-KR FLAG-PCNA with or without nocodazole (Noc) blocking. 1C and 2C represent cells containing one or two copies of each chromosome, respectively. The cells were labeled with BrdU and co-stained for BrdU incorporation (Y-axis) and DNA content (PI, X-axis). Percentages of cells in S-phase are shown. (e) ChIP analysis of RNAPIIo occupancy at FRA7K using IMMP2L3b primers for the cell samples shown in (d). For all ChIP experiments, each value represents the average value ± standard deviation calculated from triplicate qPCR reactions per one representative experiment. *p* values were calculated by t-test analysis for statistically significant differences. Only *p* values equal to or less than 0.05 are shown. Results were reproduced in at least 2 independent experiments.

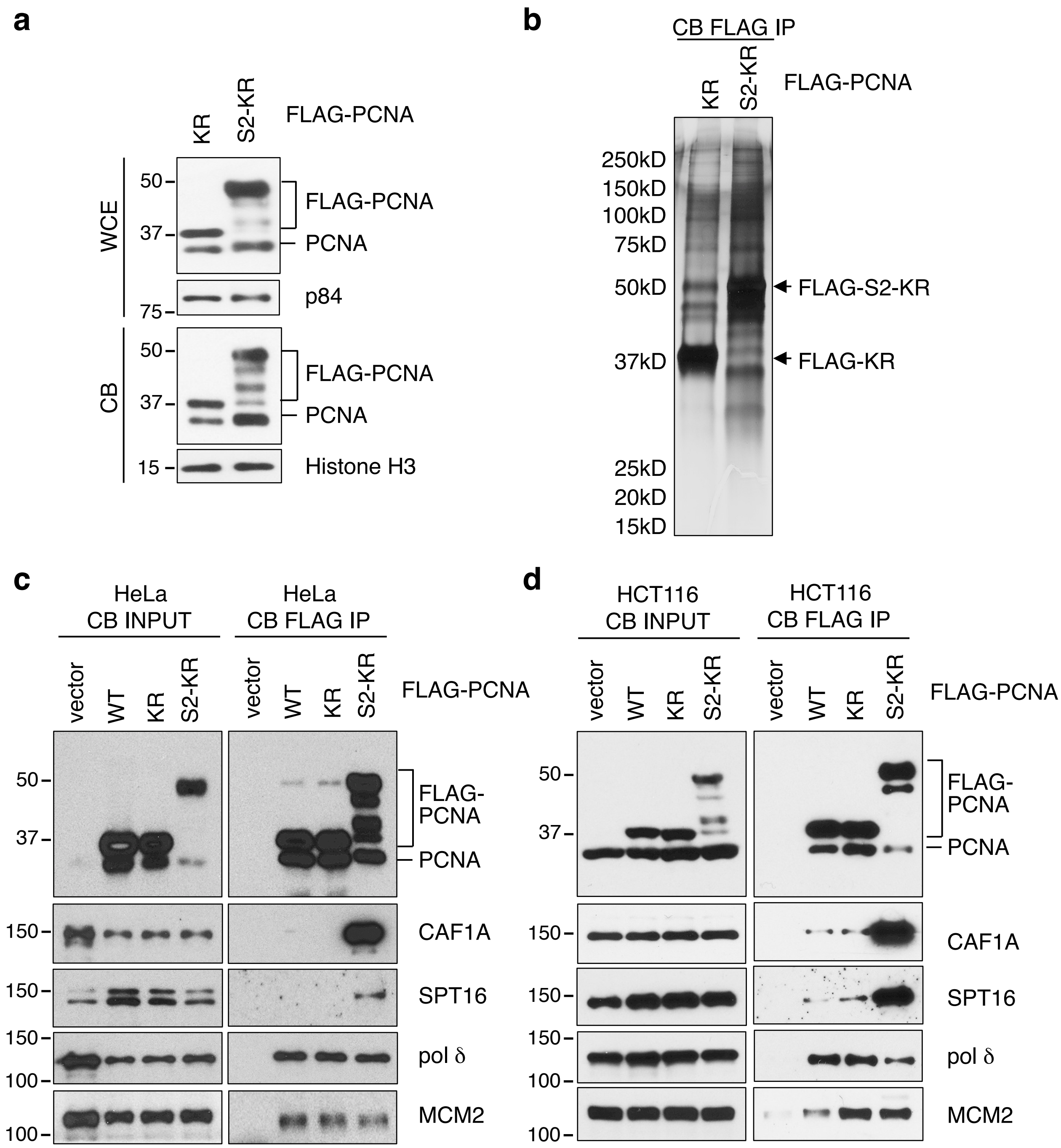

Supplementary Figure 5. Purification of SUMO2-PCNA complex from human chromatin. (a) Western blot analysis of PCNA and FLAG-PCNA in whole cell extracts (WCE) (top) and the chromatin-bound (CB) fraction (bottom) prepared from cells expressing the indicated FLAG proteins. Blots were probed using an antibody against PCNA. p84 and histone H3 are shown as loading controls. (b) Silver stained SDS-PAGE analysis of the indicated immunopurified FLAG-PCNA complexes in the CB fractions. The positions of FLAG-tagged proteins are marked with arrows. (c)-(d) Western blot analysis of the indicated proteins in the CB fractions (left) and the FLAG-PCNA complexes purified from CB fractions (right) prepared from HeLa (c) and HCT116 cells (d) expressing the indicated FLAG-tagged proteins.

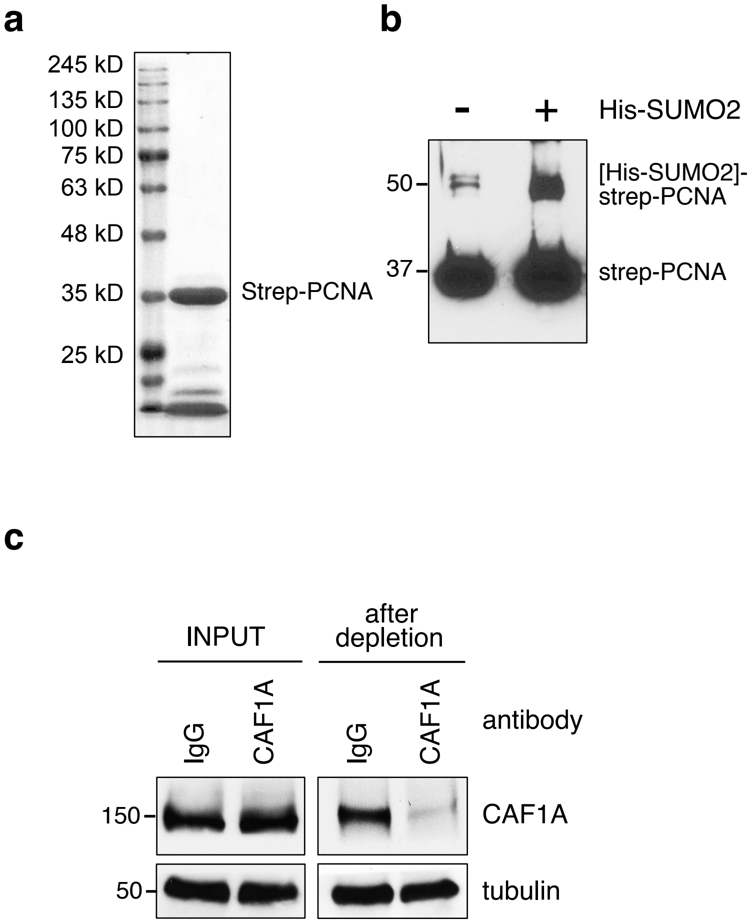

Supplementary Figure 6. ***in vitro* SUMO2 conjugation of PCNA** (a) Coomassie blue staining of purified *E. coli*-expressed Strep-PCNA. Molecular markers are shown on the left. (b) Western blot analysis of Strep-PCNA in SUMOylation reactions with or without His-SUMO2. (c) Western blot analysis of CAF1A in S2-PCNA overexpressing extracts before and after CAF1A depletion using an α-CAF1A antibody. Tubulin was used as a loading control.

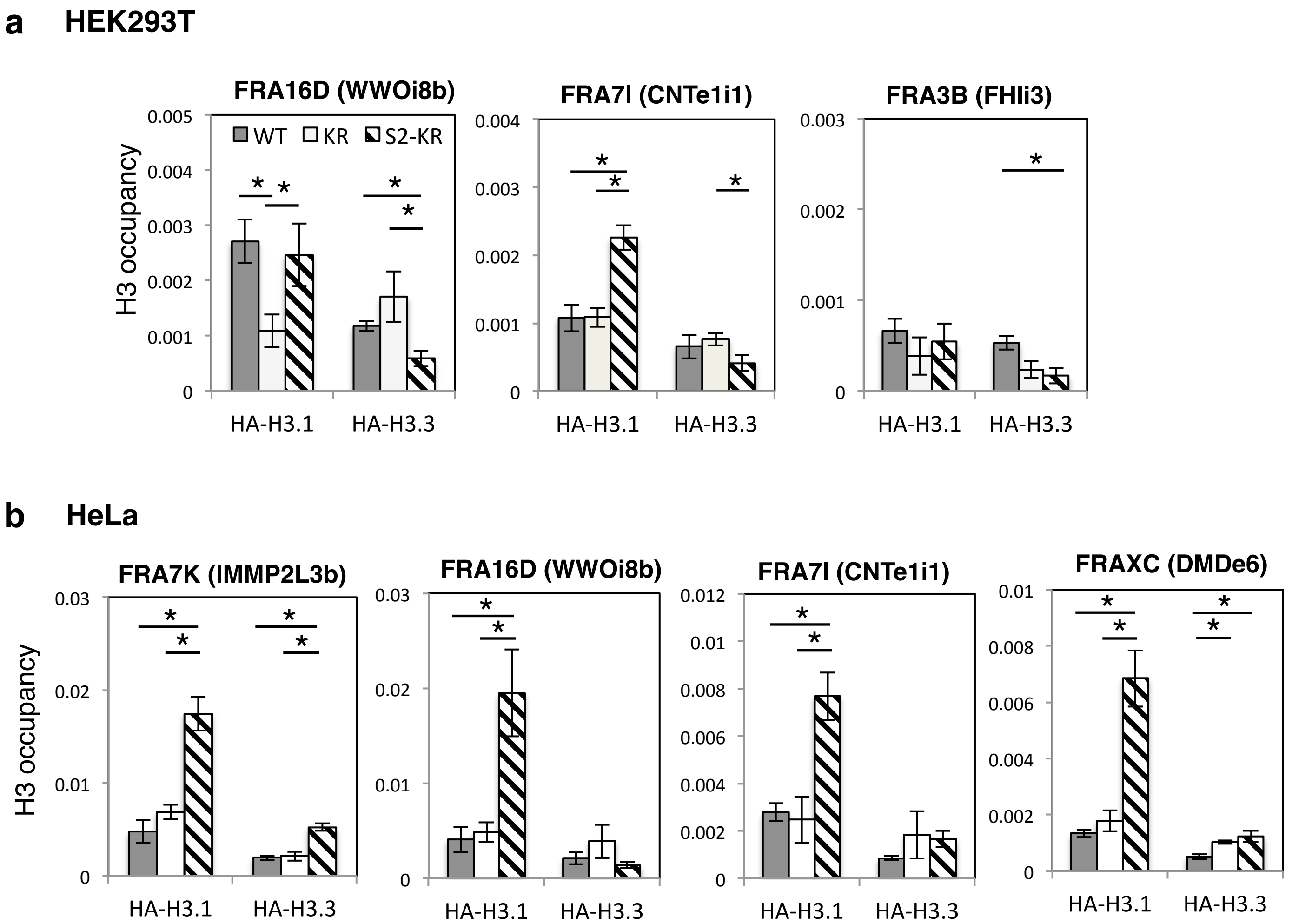

Supplementary Figure 7. Histone H3.1 and H3.3 occupancy at CFSs. ChIP analysis for HA-H3.1 and HA-3.3 occupancy at the indicated CFS gene regions in (a) HEK293T and (b) HeLa cells overexpressing the indicated FLAG-PCNA and HA-H3 proteins. For all ChIP experiments, each value represents the average value ± standard deviation calculated from triplicate qPCR reactions per one representative experiment. *p* values were calculated by t-test analysis for statistically significant differences. *p* values equal to or less than 0.05 are indicted with an asterisk (*). These results were each reproduced in at least two independent experiments.

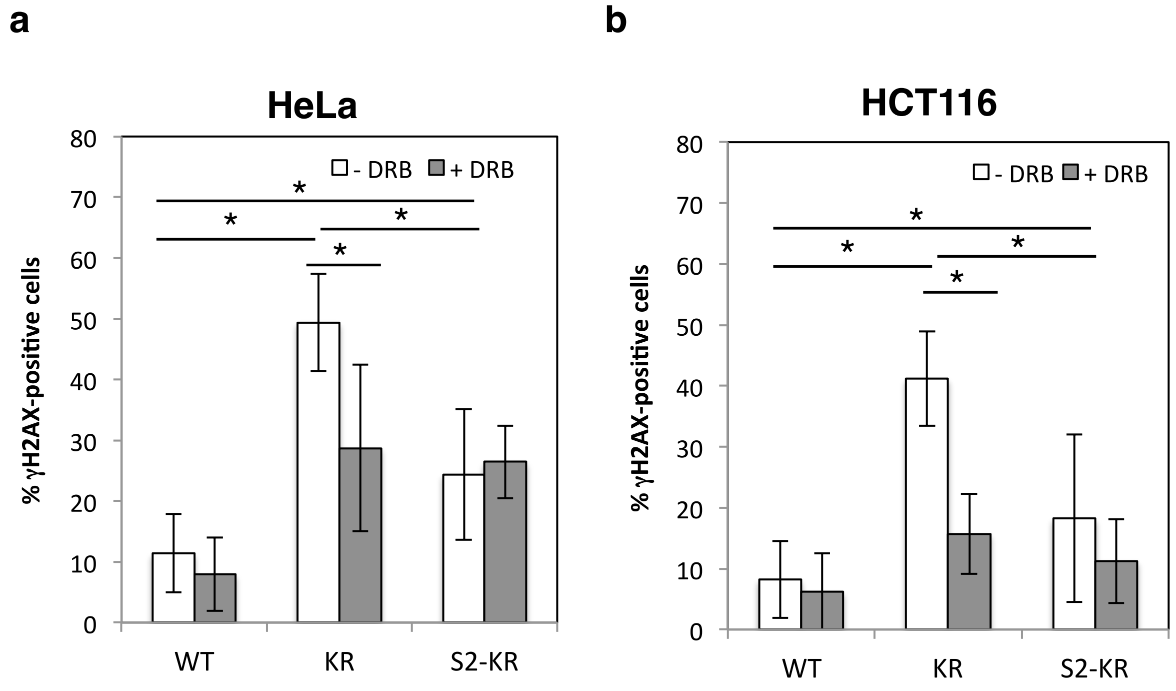

Supplementary Figure 8. SUMO2-PCNA suppresses transcription-induced DSBs. Quantification of γH2AX-positive (a) HeLa and (b) HCT116 cells overexpressing the indicated FLAG-PCNA complexes with or without DRB treatment (100 μM). Each value in the graph represents the average value ± standard deviation (n>200) per one representative experiment. Only cells with 5 or more γH2AX foci were counted as γH2AX-positive cells. *p* values were calculated by t-test analysis for statistically significant differences. *p* values equal to or less than 0.05 are indicated with an asterisk (*). These results were each reproduced in at least two independent experiments.

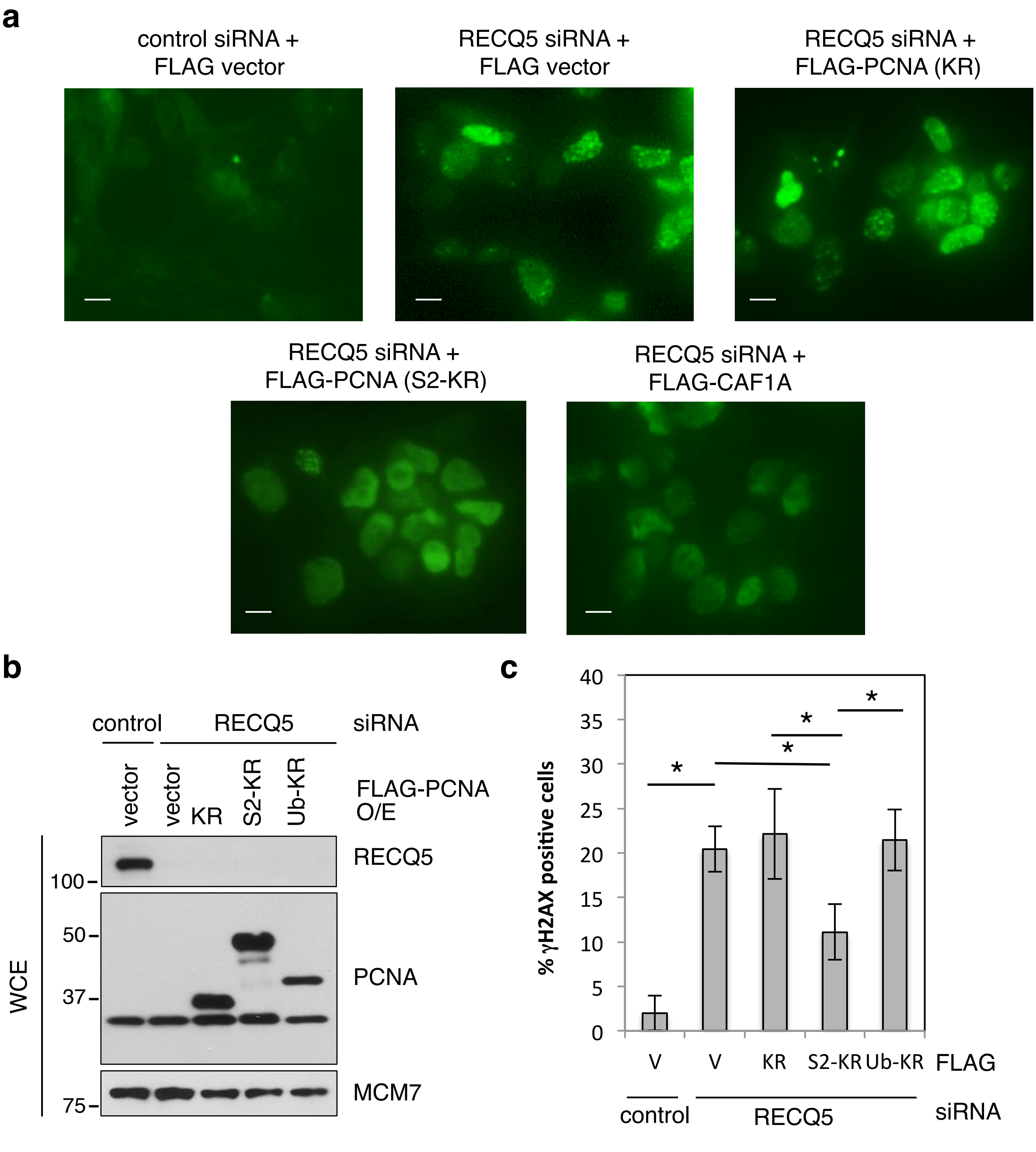

Supplementary Figure 9. SUMO2-PCNA suppresses transcription-induced DSBs in RECQ5 knockdown cells. (a) Representative fluorescence images of γH2AX foci in control or RECQ5 knockdown HEK293T cells overexpressing the indicated FLAG-PCNA or CAF1 proteins shown in Fig. 7i-j. Scale bar, 10 µm. (b) Western blot analysis of PCNA and FLAG-PCNA in whole cell extracts (WCE) prepared from control or RECQ5 knockdown cells overexpressing the indicated FLAG-PCNA proteins. The blots were probed using antibodies against PCNA and RECQ5. MCM7 was used as a loading control. (c) Quantification of γH2AX-positive cells overexpressing the indicated FLAG-PCNA complexes with or without DRB treatment (100 µM). Each value in the graph represents the average value ± standard deviation (n>200) per one representative experiment. Only cells with 5 or more γH2AX foci were counted as positive cells. *p* values were only calculated to compare RECQ5 knockdown cells with and without complementation with PCNA KR, S2-KR, and Ub-KR and with control knockdown cells. Only *p* values equal to or less than 0.05 are indicated with an asterisk (*). The result was reproduced in two independent experiments.

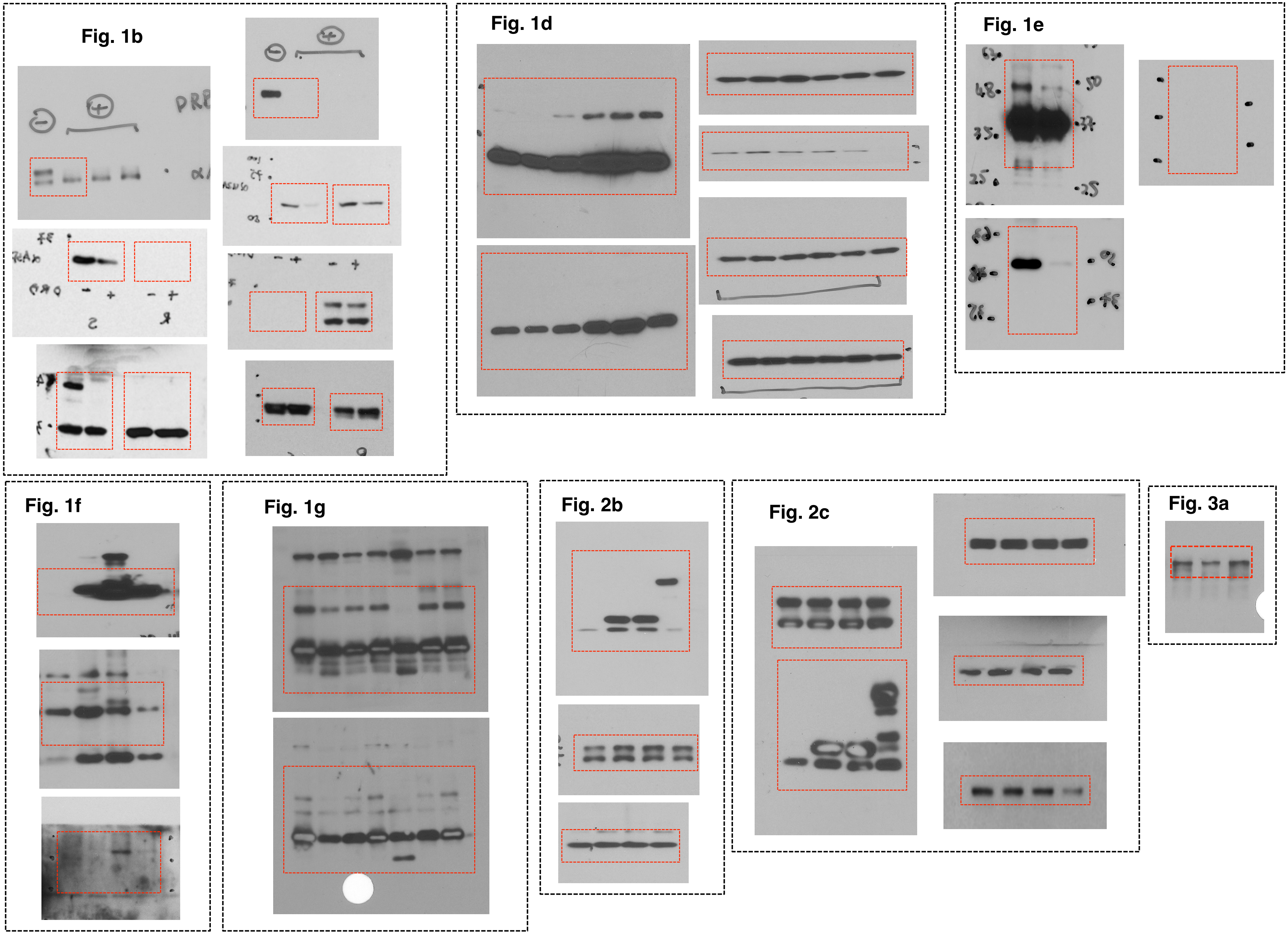

Supplementary Figure 10. uncropped images for Figures 1b, 1d, 1e, 1f, 1g, 2b, 2c and 3a.

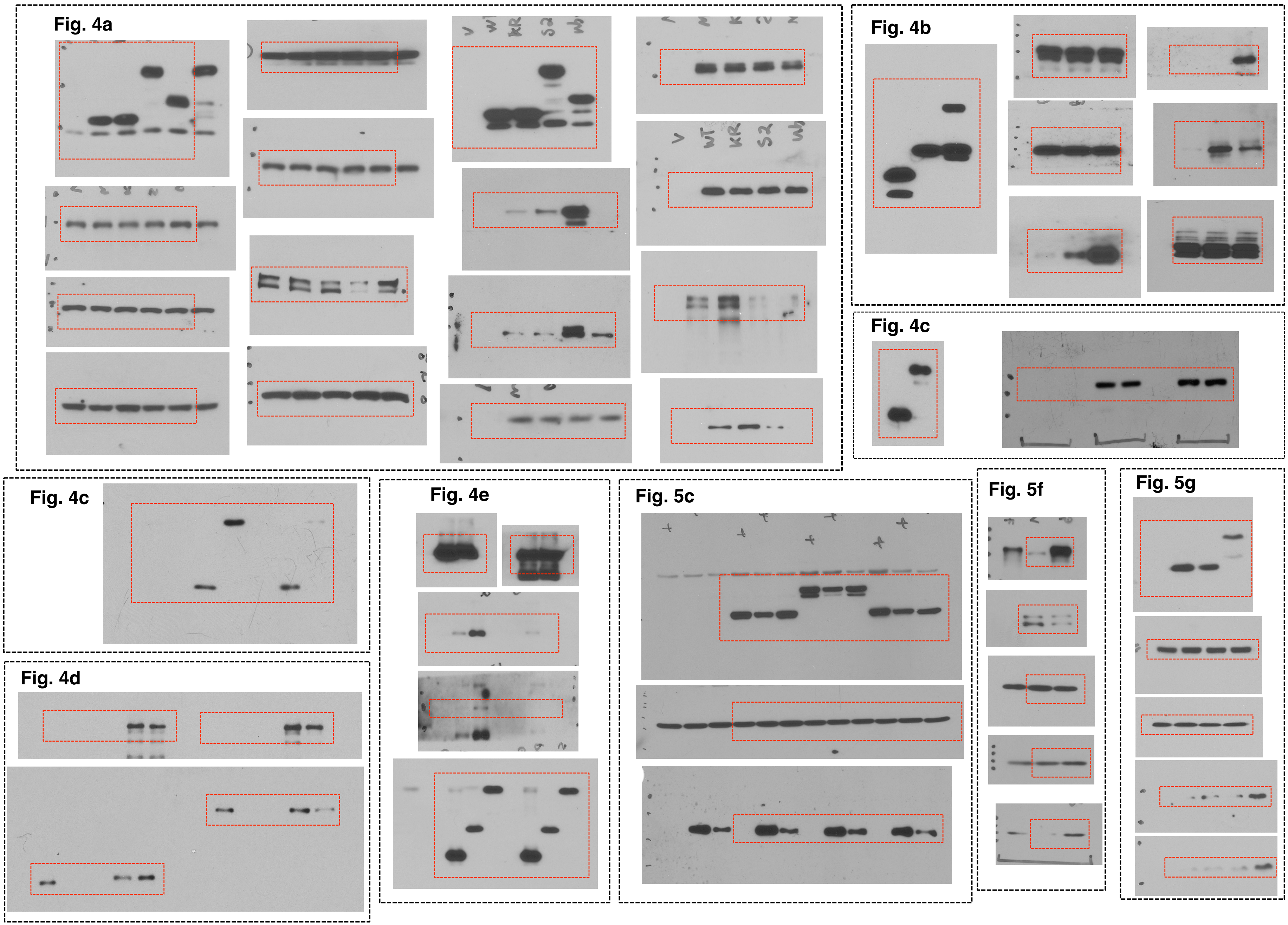

Supplementary Fig. 11. Uncropped images for Figures 4a-e, 5c, 5f and 5g.

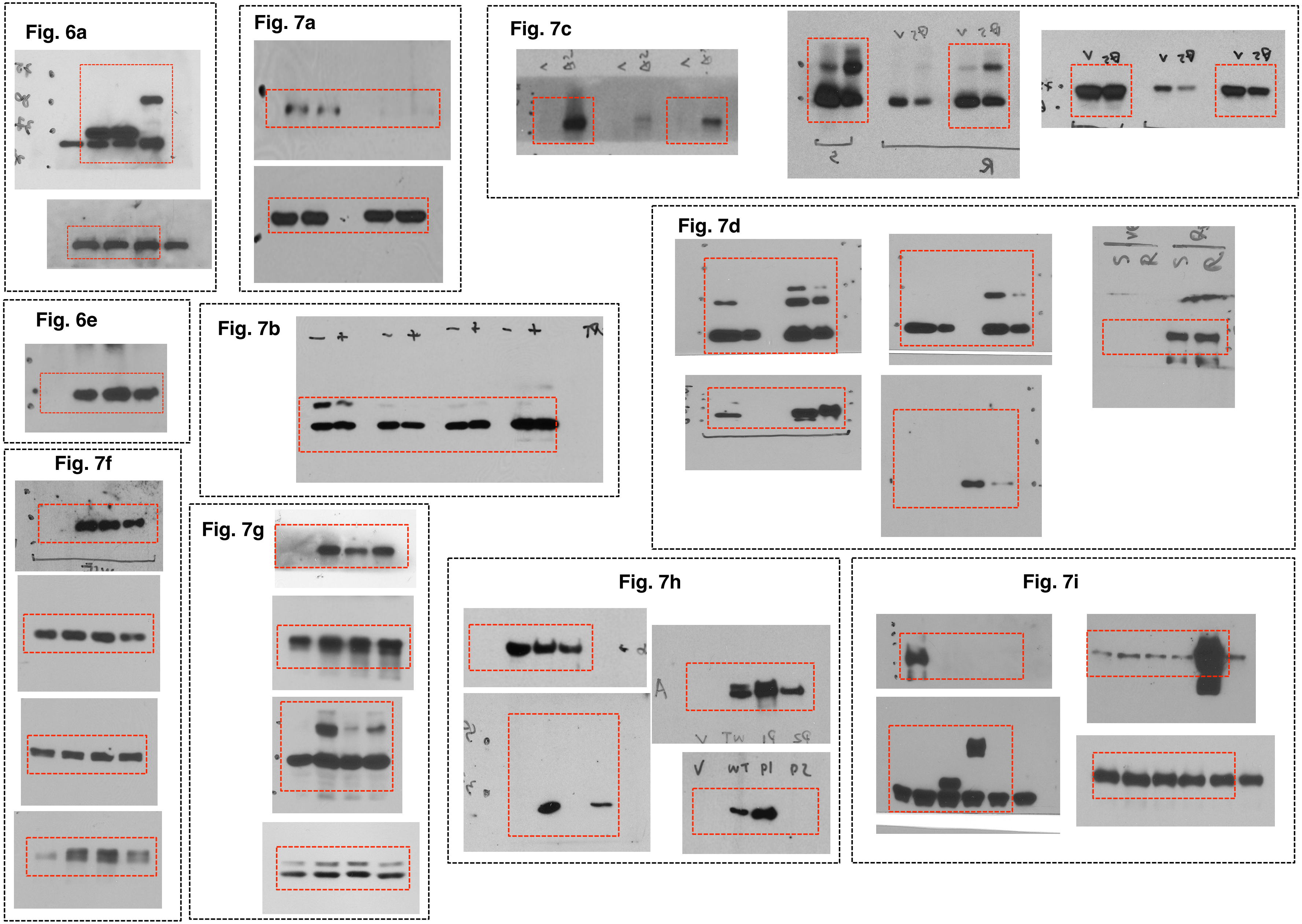

Supplementary Figure 12. Uncropped images for Figures 6a, 6e, 7a, 7b, 7c, 7d, 7f, 7g, 7h and 7i.

Supplementary Table 1: Primers for mutagenesis

PCNA K110R	GTATTTGAAGCACCAAACCAGGAGAGAGTTTCAGACTATGAAATGAAGTTG
PCNA K117R	GAGAAAGTTTCAGACTATGAAATGAGGTTGATGGATTTAGATGTTGAACAA
PCNA K138R	AGGAGTACAGCTGTGTAGTAAGGATGCCTTCTGGTGAATTTGC
PCNA K164R	TGCTGTTGTAATTTCCTGTGCAAGAGACGGAGTGAAATTTTCTGCA
PCNA K168R	TTCCTGTGCAAAAGACGGAGTGAGGTTTTCTGCAAGTGGAGAACTTGG
PCNA K254R	CTTAAAATACTACTTGGCTCCCAGGATCGAGGATGAAGAAGGATCTT
RECQ5 PIP-LA	CCACAAGGATTCTCAGAGCGCCGCCCGCGCCGCCTGCCGAAGGGTGGAAAGC
RECQ5 PIPA	GAGGCCCAGAACCTCGCCAGGCACGCCGCCCATGGCCGGGCCCG
SSRP1 SIM	CCAAACTCGCTACCACTTCGCAGCAGCAGCAGCATCCAAGGACGAGGACATTT
CAF1A SIM	ATTGGCCAGAGCACAGTCGCAGCAGCAGCAGCAGAGGACTCGAATGAGCAG

Supplementary Table 2: Primers used for ChIP qPCR

Primer name	Forward	Reverse
IMMP2L1	ctgtgtcctgaacgtgccta	tgaatcaatgaaggaacccatt
IMMP2L2	tgtgcctgtatggttcctga	ctggtcccagtttgagccta
IMMP2L3a	cctggggtgggttggaggta	tggcattcttcagcaaagaactaggg
IMMP2L3b	tgtgccactgtcccccagac	agccctcctcctggctgctt
IMMP2L5	ttgtgcctcctgatttgatg	atctggagtcccagcaagc
WWOXi4	cgcccggccttgatttttct	ggcaagcaatggaagcgtga
WWOXi5	atgtgccagtcacagtggaa	ccttgctaatggaagccaaa
WWOXi8a	gccaaaaccacctccgttgc	gccatgggacacttgggaca
WWOXi8b	cagccgcaactgttactcaa	accacaggtaagggggtaca
DMDe6	gctggattgcaacaaaccaaca	tcaaagccaggccatcagacc
CNTe1i1	agcgctcctgctgtggattg	gccgcactgaaactgctcca
FHIi3	ttgggggaacagaattcaac	catgctgcctaccttctggt
ACTB EX3	gctcagggcttcttgtcctt	tcgatggggtacttcagggt
ACTBUPS	cccatctgaaacatggggct	ggccagggaaagtgagagac
