# Supplementary material for: SUMO2 conjugation of PCNA facilitates chromatin remodeling to resolve transcription-replication conflicts
Source: Nat Commun. 2018 Jul 13;9:2706. doi: 10.1038/s41467-018-05236-y (PMC6045570; doi:10.1038/s41467-018-05236-y)
Supplement: Supplementary file 3 — Description of Additional Supplementary Files [file 41467_2018_5236_MOESM3_ESM.pdf]

Description of Additional Supplementary Files

File Name: Supplementary Data 1
Description: Lists the identity of all the proteins, number of peptides per protein, the intensity of the peptides identified in our Mass Spec analysis of the purified FLAG-PCNA KR and FLAG-SUMO2-PCNA KR (S2-KR) fusion protein complexes.
